# The Newspaper and the Medical Profession

**Published:** 1917-06

**Authors:** 


**A Monthly Review of Medicine and Surgery**

EDITOR AND PUBLISHER, DR. A. L. BENEDICT, 228 Summer St., corner of Elmwood Ave., Buffalo, (Address for all communications. Please make personal and telephone calls before 1 P. M.)

Third, in age, on the western continent. Independent, but supports professional organizations. News limited mainly to Western N. Y. and Penn.

Subscription $2.00 a year; $3.00 for two years in advance. Changes and errors in address or failure or duplication of delivery, should be reported immediately.

Yearly Volume 72 JUNE, 1917 Number 11

### The Newspaper and the Medical Profession.

A few years ago, the physician regarded the newspaper as a factor only of his extra-professional life; indeed, not a few physicians of good standing rather prided themselves on not keeping in touch with the political and other news of the day. So far as the newspaper was considered as bearing on professional interests directly, the main topic of discussion was the quack advertisement and the closely related ethical problem as to how far the social and other extra-professional activities of the physician should appear in the public press. As to the latter point, it was disputed to what extent an officious reporter had intruded into the privacy of professional practice and to what extent a physician, nominally ethical, had connived with the newspaper man to secure publicity in an underhand manner, quite analogous to that for which the quack openly paid. Of course, anything of great public interest developing in medical circles, usually found its way into print, but news of this sort was rarely accurate and usually such as involved scandal of some sort or, at least, sensationalism.

Gradually but quite rapidly, a marked change has taken place. The problem of the quack advertisement remains unsolved, except as a limited number of lay publications have voluntarily eliminated such matter and as legislation, while scarcely able to touch matters of ethics in a vital spot, has at least imposed certain requirements as to competency on all practitioners, irrespective of ethics, and certain requirements as to honesty of composition and claims, upon foods, drugs and various other articles of merchandise. As to mention of physicians, it is pretty generally agreed that social, political and other activities may be noted as for anyone else. The better class of newspapers, physicians and laymen, agree that the attendance of a physician upon a case should not be published, and that such a violation of good taste, even if not of good ethics, reacts to the detriment of the physician. The lower class of newspapers obviously do not follow standards of either ethics or taste, the lower class of physicians may still court such publicity and it may do them good—in the business sense—among the lower class of the laity. At any rate, it is pretty generally agreed that about all has been said upon these subjects that can be said.

Newspapers of the present publish medical news as of other sciences or organizations, and, on the whole, without attempt at sensationalism and with a genuine spirit of giving to the public matter which is of real public interest. Errors of general conception, not to mention mistakes in technicalities, often occur, but probably no more frequently than in reports of electric, geologic or other scientific news. The fact that every community of any considerable size has a daily newspaper, and that cooperation has secured a very perfect means of securing news from the entire world with surprising promptness and accuracy, renders the invasion of the medical field by the public press, valuable even to the medical profession. For example, the epidemic of poliomyelitis has been put before the medical profession in a way that could not have been accomplished by the medical press, except, of course with regard to technical details. Notices of medical meetings are also published with accuracy, instead of the occasional haphazard announcement that Dr. John Smith, of our city, was elected vice-president of a society of doctors held in some other city. Discoveries in medicine are also announced more promptly than and, on the whole, with quite as good judgement as to their scientific importance as in medical journals. The organized profession and its representatives in sanitary official positions and in private organizations to secure hygienic and sociologic reforms, regularly employ the public press to reach the laity and, by reiterated advice and information, the laity has become educated to a knowledge of the fundamental principles of hygiene and sanitation and indeed of medical science itself, fairly comparable to what it possesses of any other art or science. Obviously, the individual layman, according to his individual intelligence, has very different degrees of appreciation of matters bordering on any form of technical knowledge but, as a profession, we must acknowledge a very general increase of intelligence in the cooperation of the laity.

It may be claimed that the curtailment of the more flagrant forms of quackery is due very largely to the increasing attention of the daily press to medical matters, and that the news items thus published go a long way toward counteracting the harm of quack advertisements in adjacent columns.

Any innovation arouses criticism. It has been charged that public officials, officers of philanthropic and educational organizations, private physicians writing upon medical and hygienic subjects in lay periodicals, discoverers and those in charge of society programs, have sought personal glory or indirect financial gain under cover of reaching the laity through the popular press. It has been seriously proposed to prohibit the publication of medical information for the laity, except by duly appointed representatives of the profession, and equally seriously objected that such a limitation was merely an attempt to monopolize publicity by the elite. We have already discussed the special issue as to whether a prominent man should enjoy an ethical license not granted to the profession generally and, while we have not indulged in attacks on those who have apparently assumed such license, we have declared unequivocally in favor of the principle that an ethical rule should apply impartially to all. As in the case of the minor problems of the past, these problems are gradually working their own solution by the establishments of canons of good taste and good sense. Most of the profession seem to feel that whatever glory or gain incidentally attaches to the sincere performance of any public service, either in an official capacity or as a matter of public spirit, is allowable—and this sentiment is quite in accord with the ancient prohibition of muzzling the ox that treads out the grain. This sentiment does not prevent the exploitation of either public service or private philanthropy by individuals but, in the long run, insincerity is a boomerang and the man who seeks notoriety, though successful, finds that he has got just what he sought and that very few confuse it with fame.

As in other instances, standards of ethics, or rather rules of conduct, vary with time and locality. For example, in Paris, medical journals have for some years been regularly on sale at the public kiosks. Here, while recognizing that a medical journal must be to some degree accessible to such members of the laity as may have a legitimate interest in it, the general sale of medical journals to the laity is regarded unfavorably. A recent issue of the Bulletin of the Society of Medicine of Paris, announces that the Temps, a Paris newspaper will publish the transactions of the Society providing 50 of its members subscribe—subscriptions being received for as short a period as three months. Incidentally, it may be noted that Paris newspapers are much smaller and more expensive than American dailies, though probably quite as valuable on account of condensation of news and the much smaller average space of an advertisement. For example, the Temps has a regular annual subscription rate of $11 a year, but it is offered to members of the Society at $8.

A suggestion of this sort, made without apology, and certainly with no consciousness on the part of the editors of the Bulletin that critics might attribute improper motives to them, but on the contrary with a frank appeal to its readers to benefit by a favor which will secure a valuable publicity to the labors of the Society, seems radical to us, but it is no more radical than the present accepted customs of our own country would have seemed to our predecessors. It should, therefore, be considered without prejudice in either direction, as an issue that may ultimately arise in our own country, and that may solve many financial and administrative problems with which medical journalism is confronted.

Whether this or any other particular issue arises, and however any such issue may be decided, the general fact must be clearly recognized that, spontaneously, without premeditation on the part of either professional or public journalism, and almost without realization on the part of either, these two lines of publicity have been approaching each other. Neither can ignore the existence of mutual interests and the possibilities of mutual benefits, not to mention the benefits accruing to the public. A return to the old conception that the newspaper or any other periodical had no concern with medicine, is out of the question. On the other hand, the general ethical principles on which this conception was based, must be preserved, however much the rules of conduct depending on them may be relaxed. As has been implied, the problems which now seem difficult will probably solve themselves gradually. It does not, therefore, seem advisable to attempt to form methods of solution which the future may show to be impracticable and fallacious, just as the prohibitive methods which our predecessors would undoubtedly have formulated if they could have foreseen present conditions, would now be recognized as unwise. But the main fact of the close relations of medicine to the public press must be kept in mind and all who have the influence to direct those relations into proper channels, should be on their guard to prevent unworthy exploitation of publicity, without obstructing natural currents toward the accomplishment of great public good.

